# Effects of bioinsecticide exposure route on aphids and their natural enemies in oilseed rape

**DOI:** 10.1002/ps.70518

**Published:** 2026-01-13

**Authors:** Aimee J. Tonks, Tom W. Pope, Simon Cooper, Joe M. Roberts

**Affiliations:** ^1^ Entomology Group, Centre for Crop and Environmental Science, Agriculture and Environment Department, Harper Adams University Edgmond UK; ^2^ Harper Institute of Technology, Engineering Department, Harper Adams University Edgmond UK

**Keywords:** aphid parasitoids, biological control, integrated pest management, lacewings, non‐target effects

## Abstract

**BACKGROUND:**

*Myzus persicae* Sulzer and *Brevicoryne brassicae* L. are economically important aphid pests of oilseed rape (OSR) and the primary vectors of turnip yellows virus. Control options are constrained for many aphid pests due to pest resistance to synthetic chemical insecticide active ingredients or their withdrawal from market. Physically acting bioinsecticides may offer an alternative control option, yet their efficacy against aphids and compatibility with natural enemies outside of horticultural production systems is poorly understood. Three bioinsecticides based on fatty acids, silicone polymers or surfactants were tested against two economically important aphid species and non‐target effects on their natural enemies, *Diaeretiella rapae* M'Intosh adults and mummies as well as *Chrysoperla carnea* Stephens larvae, were also assessed.

**RESULTS:**

Under direct exposure, fatty acids, silicone polymers and surfactants all caused aphid mortality (*B.* brassicae 90–56%, *M. persicae* 63–20%) within 72 h. *Diaeretiella rapae* mortality was 100% 24 h after exposure to fatty acids and silicone polymers while *Chyrsoperla carnea* mortality was 66% and 100%, respectively. Residual exposure caused limited mortality in aphids (*M. persicae* 0%, *B. brassicae* ≤10%) and natural enemies (*D. rapae* ≤33%, *C. carnea* ≤13%) compared to the sulfoxaflor synthetic chemical insecticide control (66–100%).

**CONCLUSION:**

Fatty acids and silicone polymers significantly reduced numbers of aphids but showed acute toxicity to parasitoids under direct exposure. Their lack of residual activity means that precise targeted application to pest populations is required but allows natural enemy populations to recolonise treated areas rapidly. These bioinsecticides may provide supplementary control within OSR integrated pest management programmes when applied strategically. © 2026 The Author(s). *Pest Management Science* published by John Wiley & Sons Ltd on behalf of Society of Chemical Industry.

## Graphical abstract

Fatty acids, silicone polymers and surfactants reduced aphid numbers but caused natural enemy mortality under direct exposure, while minimal residual activity suggests potential for targeted, compatible use in integrated pest management.

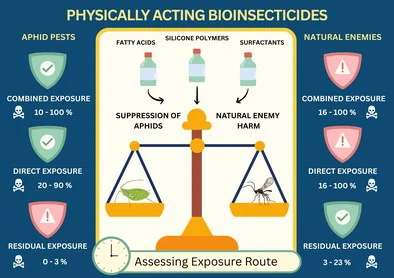

## 1 INTRODUCTION

Winter oilseed rape (OSR; *Brassica napus* L.) is a widely cultivated break crop in UK arable rotations grown for its agronomic benefits and the production of oil‐rich seed.^1^ However, pest pressure from virus vectoring aphids, such as the peach potato aphid (*Myzus persicae* Sulzer, Hemiptera: Aphididae) and mealy cabbage aphid (*Brevicoryne brassicae* L., Hemiptera: Aphididae), presents a significant barrier to achieving economically viable yields through direct feeding and by acting as virus vectors. In UK winter OSR, autumn aphid populations vector turnip yellows virus during crop establishment, with even low aphid densities capable of causing yield losses exceeding 30% in subsequent seasons while spring aphid populations can reach damaging densities on flowering crops, although economic thresholds are poorly defined for this growth stage.^2^ Integrated pest management (IPM) provides a framework for the sustainable control of pest populations in cropping systems by combining multiple compatible strategies to reduce overreliance on a single control method.^3–5^ In the context of UK arable agriculture, particularly in winter OSR, the identification and implementation of diverse pest management tools has become increasingly critical in achieving economic viability. An over‐reliance on use of synthetic chemistry has accelerated the development of resistance in pest populations, which has been further compounded by a diminishing active ingredient portfolio due to product withdrawals linked to environmental and human health concerns.^6^ This has driven an urgent need for novel, effective alternatives.^7^ As the sector transitions towards more integrated and sustainable pest management strategies, it is essential that new tools are developed and deployed with a clear understanding of their ecological impacts, particularly their effects on non‐target organisms, and that they are used in a manner compatible with existing biological control agents and the core principles of IPM.^8^

Natural enemies, including hymenopteran parasitoids and generalist predators, play a key role in IPM by regulating pest populations in OSR^3^, ^4^ through their parasitism and predation behaviours, collectively known as biological control.^5^ *Diaeretiella rapae* M'Intosh (Hymenoptera: Braconidae) is a specialised aphid parasitoid commonly found in OSR crops that overwinters as diapausing mummies in field margins, with adult emergence and host‐searching activity occurring from March through October, peaking during spring aphid population growth.^6^ Generalist predators such as the green lacewing larvae *Chrysoperla carnea* Stephens (Neuroptera: Chrysopidae) also contribute to aphid suppression, with adults overwintering in field margins and found in UK OSR crops from April onwards.^7^, ^8^ The efficacy of these natural enemies is dependent on their survival within cropping systems and the temporal overlap between pest and natural enemy activity creates potential for non‐target impacts if plant protection products are applied without consideration of organism phenology. Historically, synthetic chemical insecticides, such as neonicotinoids and pyrethroids, have been deployed as prophylactic treatments in OSR.^9^, ^10^ Although often effective in the short term, this approach can lead to natural enemy mortality,^11^ disruption of prey/host seeking or reproductive success through sub‐lethal effects,^12^, ^13^ and the wider destabilisation of ecological networks.^14^ This can result in secondary pest outbreaks, diminished crop protection services and potential for increased reliance on further synthetic chemical intervention.^15^, ^16^, ^17^ Regulatory authorities and stakeholders are increasingly aware of the need to identify and develop new pest management tools.^18^ There is a growing consensus that future pest management tools must not only be effective and economically viable but also align with the principles of environmental stewardship and ecological compatibility.^19^, ^20^ As a result, bioinsecticides, naturally derived plant protection products, have gained attention for their potential to deliver pest suppression with reduced environmental and human health impacts compared to synthetic chemical ones.^21^, ^22^, ^23^, ^24^ Within the broader category of bioinsecticides, physically acting products represent a distinct subset whose mode of action relies on physical rather than biochemical interference with the pest.^25^ The bioinsecticides tested in this study represent three distinct modes of physical action marketed as alternatives to synthetic chemical insecticides.^26^, ^27^, ^28^ Fatty acid‐based products contain saturated and unsaturated fatty acids, primarily C16–C18 chains, which are reported to disrupt insect cuticle integrity and may interfere with cellular respiration and acetylcholinesterase inhibition.^29^ These products have demonstrated efficacy against soft‐bodied insects in horticultural applications and are currently registered for use in protected cropping systems. Silicone polymer‐based products utilise trisiloxane surfactants that reduce surface tension, causing suffocation through spiracle blockage and potentially disrupting cuticular waterproofing.^30^ Despite their use in agriculture, concerns have been raised regarding their effects on pollinators and beneficial arthropods.^31^ Surfactant‐based bioinsecticides employ detergent‐like compounds that solubilise cuticular lipids and may cause cell membrane disruption.^32^ While these products are marketed as ‘physically acting’ with minimal environmental persistence, field efficacy data against aphid populations in outdoor cropping systems remain limited, and their compatibility with biological control agents common in UK arable systems has not been evaluated.

This study evaluated three commercially available bioinsecticides against two economically important aphid species (*M. persicae* and *B. brassicae*) and two of their key natural enemies (*D. rapae* and *C. carnea*) under three exposure scenarios: (i) combined exposure (direct spray contact with insects on treated leaves), (ii) insect‐only exposure (direct spray contact with insects subsequently transferred to untreated leaves) and (iii) residual exposure (insects placed on treated leaves after spray residues had dried). By comparing mortality across these exposure routes, we aimed to determine whether bioinsecticide efficacy depends primarily on direct contact or persists as residual toxicity, and whether this exposure‐response relationship differs between pest and natural enemy species.

## 2 MATERIALS AND METHODS

### 2.1 Plants and insects

#### 2.1.1 Plant cultivation

OSR (*var*. Duplo) seeds were individually sown into coir compost plugs (30 × 38 mm; Jiffy, Lindtsedijk, The Netherlands) and grown within a controlled environment room (Weiss Technik UK Ltd, Loughborough, UK) at 20 °C and 60% relative humidity with a 16 h:8 h (light: dark) photoperiod until reaching Biologische Bundesanstalt, Bundessortenamt und CHemical Industry (BBCH) growth stage 10 (cotyledons completely unfolded).^33^ Seedlings were then transplanted into 9‐cm pots containing Levington Advance Pot and Bedding Medium Nutrient Professional Growing Media (M2) (Levington, Frimley, UK) and maintained in mesh cages (60 × 60 × 60 cm; BugDorm, MegaView Science Co., Taichung, Taiwan) in the glasshouse prior to use in aphid stock cultures, standardised aphid cohorts and experiments.

#### 2.1.2 Stock aphid culturing

Stock cultures of *Myzus persicae* Sulzer (Hemiptera: Aphididae) and *Brevicoryne brassicae* L. (Hemiptera: Aphididae) were maintained on OSR (*var*. Duplo) seedlings at BBCH growth stage 12 (second true leaf unfolded) to 14 (fourth true leaf unfolded).^33^ These were individually housed in mesh cages (47 × 47 × 47 cm; BugDorm, MegaView Science Co.) within a controlled environment room (Weiss Technik UK Ltd) at 18 °C and 60% relative humidity with a 16 h:8 h (light: dark) photoperiod. Fresh plant material was provided weekly and watered *ad libitum* so that the compost remained moist below a surface depth of 2 cm.

#### 2.1.3 Standardised aphid cohorts

Age‐synchronised cohorts of third instar, representing a developmentally vulnerable but mobile stage, *M. persicae* and *B. brassicae* were used in all experiments. These cohorts were prepared by transferring 50 adult apterous aphids of a single species from the primary culture to an uninfested OSR seedling at BBCH growth stage 12 using a 000 paintbrush. Aphids and their host plant were housed in a fine mesh insect cage (30 × 30 × 30 cm; BugDorm, MegaView Science Co.) for 24 h. Adult apterous aphids were removed from the plant using a 000 paintbrush after this period, leaving a population of age‐synchronised aphid nymphs. Aphid nymphs were maintained on the OSR plant in the temperature‐controlled environment room at 18 °C and 60% relative humidity with a 16 h:8 h (light:dark) photoperiod until they reached third instar. This was determined by monitoring nymphs daily and removing exuviae to identify aphid instar moults. The time taken for nymphs to develop to third instar was approximately 7 days under the controlled conditions. On reaching third instar, the nymphs were removed from the age‐standardised cohort and used in experiments.

#### 2.1.4 Stock parasitoid culturing

*Diaeretiella rapae* M'Intosh (Hymenoptera: Braconidae) were maintained on 10% honey‐water solution supplied twice‐weekly on soaked cotton wool. Mixed age *M. persicae* and *B. brassicae* on OSR were supplied weekly from the stock aphid cultures to support parasitism. The parasitoid stock culture was housed in a mesh cage (47 × 47 × 47 cm; BugDorm, MegaView Science Co.) within a controlled environment room (Weiss Technik UK Ltd) at 20 °C and 60% relative humidity with a 16 h:8 h (light:dark) photoperiod.

#### 2.1.5 Standardised parasitoid cohorts

Age‐synchronised cohorts of *D. rapae* were used in all experiments. These cohorts were prepared by transferring 30 adult *D. rapae* from the primary culture to OSR seedlings BBCH growth stage 12–14) containing 300 fixed age third‐instar *M. persicae*. Female adult parasitoids were collected from the stock culture using a glass aspirator (75 × 25 mm; Watkins & Doncaster, Leominster, UK) and CO_2_ was used to temporarily immobilise the wasps for transfer into the new culture. Adults were visually sexed, with females identified by presence of the ovipositor sheath.^34^ Parasitoids and the aphid infested host plant were housed in a fine mesh insect cage (30 × 30 × 30 cm; BugDorm, MegaView Science Co.) for 24 h. Adult parasitoids were removed from the culture using a glass aspirator after this period, leaving a population of age‐synchronised parasitised aphid nymphs. Parasitised aphid nymphs were maintained on the OSR plant in the temperature‐controlled environment room at 20 °C and 60% relative humidity with a 16 h:8 h (light:dark) photoperiod until mummification. Mummies used in experiments were standardised to 7 days post‐parasitism, corresponding to the non‐feeding, protected pupal stage, Adults used in experiments were standardised to 2 days post‐emergence and maintained on 10% honey‐water solution on soaked cotton wool for 2 days prior to use.

#### 2.1.6 *Chrysoperla carnea* larvae

*Chrysoperla carnea* (Neuroptera: Chrysopidae) larvae, representing the functionally predatory stage most relevant to aphid predation and foliar exposure, were purchased from Bioline Agrosciences (Little Clacton, UK) and were supplied as early mixed instars. They were maintained in the temperature‐controlled environment room at 20 °C and 60% relative humidity with a 16 h:8 h (light:dark) photoperiod and monitored daily until moulting into third instar. Individuals were selected for experimentation based on moulting events and visual confirmation of body size (with body length exceeding a threshold of 6.1 mm following a moult event^35^) with feeding standardised by providing *ad libitum* third‐instar *M. persicae* for 24 h prior to use. These procedures were designed to minimise variation in age and nutritional condition across replicates and to account for inherent variability in externally purchased populations.

### 2.2 Containment arenas

#### 2.2.1 Aphid containment

For all experiments, aphids were individually maintained in triple vented Petri dishes (Ø = 35 mm; Greiner Bio‐One Ltd, Gloucestershire, UK). Each Petri dish was adapted to create a 20 mm ventilation hole in the lid using a Dremel rotary multi‐tool (Robert Bosch UK Holdings Ltd, Uxbridge, UK) and covered by gluing 28‐gauge voile mesh on the lid. The base of the Petri dishes contained 1% water bacteriological agar (Agar Technical No.2, Oxoid UK, Cheshire, UK) poured to a depth of 5 mm. Leaf discs were cut from OSR (*Brassica napus* var Duplo) plants at BBCH growth stage 13 using a 30‐mm diameter wad punch piece (RS Components Ltd, Corby, UK), avoiding the leaf's central midrib. Each Petri dish contained a leaf disc, placed adaxial surface down on to the water agar to maintain moisture, exposing the abaxial surface on which aphids naturally reside and feed, and a single third‐instar aphid (*M. persicae* or *B. brassicae*) transferred using a 000 paintbrush.

#### 2.2.2 Natural enemy containment

For all experiments, *D. rapae* mummies, adults and *C. carnea* larvae were maintained individually in 1‐mL Eppendorf tubes with a pin‐hole ventilated lid (Fig. 1). Each Eppendorf tube contained 0.2 mL of 1% bacteriological water agar (Agar Technical No.2, Oxoid UK) set to one side. Leaf discs were cut from OSR (*Brassica napus* var Duplo) plants at BBCH growth stage 13 using a 10‐mm diameter wad punch piece (RS Components Ltd), avoiding the leaf's central midrib. Each Eppendorf tube contained a leaf disc, placed adaxial surface down on to the water agar around the side of the tube to maintain moisture, and a single natural enemy transferred using a 000 paintbrush. For treatments containing parasitoid adults, cotton wool soaked in 0.1 mL of 10% honey‐water was also included in the bottom of the tube. *Chrysoperla carnea* larvae were fed *ad libitum* third‐instar *M. persicae* for 24 h prior to treatment. Prey was withheld during assays to prevent larvae from ingesting aphids contaminated with bioinsecticide residues. For treatments containing parasitoid mummies, the Eppendorf tubes remained empty except for the treated mummy. Assay formats were tailored to the biological requirements of each taxon. Natural enemies were considerably more mobile than aphids, with adult parasitoids capable of flight and *C. carnea* larvae moving readily when disturbed, making repeated opening of Petri dishes for mortality assessments impractical and increasing the risk of escape or handling effects. In contrast, aphids were comparatively sedentary under assay conditions and required close inspection to reliably distinguish mortality from immobility. Pest and natural enemy assays were therefore conducted as independent experiments rather than for direct comparison.

**Figure 1:**
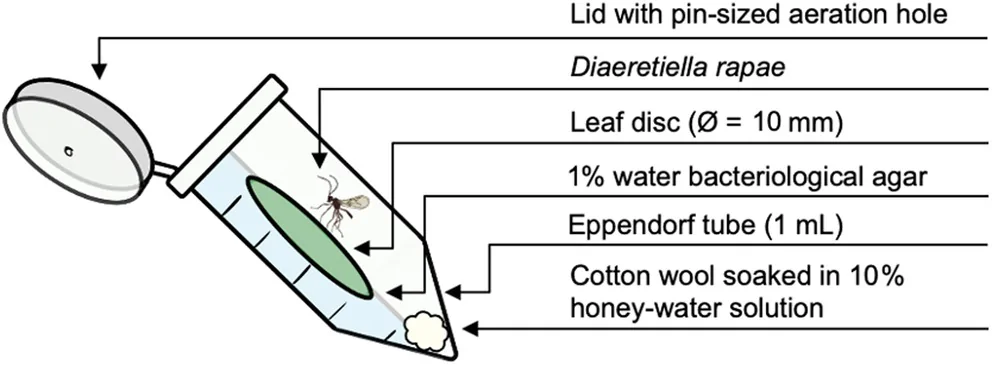
Containment of *Diaeretiella rapae* for exposure method assays.

### 2.3 Treatment and application

All experimental treatments were prepared in accordance with the concentration recommended on the product label (Table 1) and applied using a Potter Spray Tower (Burkard Manufacturing Co Ltd, Hertfordshire, UK) at a pressure of 69 kPa.^36^, ^37^, ^38^, ^39^ All products were diluted using tap water (pH 7‐8), selected to reflect the water quality typically used in commercial growing systems. Using a 5‐mL syringe, 3 mL of diluted product was transferred into the loading tube of the Potter Spray Tower and applied as required for each experiment. The volume of diluted product used (3 mL) was determined using water‐sensitive paper as the minimum volume required to achieve run‐off. All water‐sensitive paper slips were scanned at 600 dpi (dots per inch) and analysed using ImageJ software (version 1.54f, National Institutes of Health, Bethesda, Maryland, USA). A colour threshold was applied to isolate areas of water contact, followed by particle analysis to quantify the surface area covered. A volume of 3 mL applied at 0.69 bar achieved 98.3% surface coverage equivalent to the highest observed values while avoiding overapplication. This pressure–volume combination was therefore selected for all subsequent experimental applications to ensure consistent and uniform deposition without excessive run‐off. Between treatments, the Potter Spray Tower was cleaned using a triple rinse method of 4 mL water, followed by 4 mL of 70% ethanol and a further 4 mL of water before being dried using paper towel. Aphid assays for both species were conducted concurrently over 3 consecutive days, with 10 replicates per treatment completed per day (*n* = 180). Assays involving natural enemies were conducted separately for each species and life stage on different days, also over 3‐day blocks with 10 replicates per treatment per day. Within each day, treatments were applied in a randomised order, selected using a random number generator in R (v4.3.1;^40^ For each assay, all containment arenas were arranged in a Latin square design accounting for each treatment and block day and maintained in a controlled environment room at 20°C and 60% relative humidity with a 16 h:8 h (light:dark) photoperiod for 3 days.

**Table 1:** Treatments used to assess the efficacy of physically acting bioinsecticides unsaturated carboxylic acids, silicone polymers and sodium lauryl ether sulphate

Treatment	Concentration (%)*	Active ingredient	Mode of action	Manufacturer
Untreated (negative control)	NA	NA	NA	NA
Water (negative control)	NA	NA	NA	NA
FLiPPER (MAPP reference 19 154)	1.6	Fatty acids, including unsaturated carboxylic acids (C7–C20)	Cuticular disruptor	Bayer Crop Science
ProTAC SF	0.125	Silicone polymers, siloxanes and organic antioxidants	Three‐dimensional immobilising polymer network structure	Biobest Group
Fizimite	0.1	Surfactants, including sodium lauryl ether sulphate	Immobilisation	Russell IPM Ltd
Sequoia (positive control)	0.1	Sulfoxaflor	Nicotinic acetylcholine receptor disruptor	Corteva Agriscience
(MAPP reference 18 938)				

* As per recommended concentration on the manufacturer label.

Sulfoxaflor was included as a positive control due to its common use in protected horticulture, where the bioinsectides assessed in this study are predominantly deployed, and was used to confirm the susceptibility of the aphid populations.

### 2.4 Exposure method assays

Three laboratory assays were completed to determine the effect of the physically acting bioinsecticide formulations against the aphid pests *M. persicae* and *B. brassicae*, and the natural enemies *D. rapae* mummies and adults, and *C. carnea* larvae under different exposure routes.

In each assay for each species, survival was assessed at 30‐min intervals for an initial period of 5 h post spray application. Further assessments were then carried out at 24‐h intervals for a total period of 72 h post application for aphids and a single assessment at 24 h for natural enemies. At the point of assessment, each containment arena (Petri dish or Eppendorf tube) was placed under a stereo microscope (Microtec HM‐3, Tec Microscopes Ltd, Somerset, UK) where the insects were exposed to a mechanical stimulus consisting of being gently touched with a 000 paintbrush. Survival was recorded when individuals showed any movement of antennae, legs or body segments in response to the mechanical stimulus; absence of movement was scored as dead. This protocol was adapted from the Insecticide Resistance Action Committee Susceptibility Test Method 019.^41^ Emergence of *D. rapae* adults from aphid mummies was recorded 14 days post spray application, with full body emergence from the mummy being classed as successful emergence.

#### 2.4.1 Combined exposure

A combined exposure assay was carried out to assess the effect of the three physically acting bioinsecticides against the pests *M. persicae*, *B. brassicae* and the natural enemies *D. rapae* adults and mummies, and *C. carnea* larvae when applied directly to both the insect and localised leaf area. In assays containing *M. persicae* and *B. brassicae*, treatments were applied to the entire containment including the aphid. In assays containing natural enemies, following application, treated leaves were transferred to the Eppendorf arena whilst still wet using storkbill fine‐pointed forceps (Watkins & Doncaster, Herefordshire, UK), cleaned with 70% ethanol followed by a water rinse between each treatment. A single treated insect was then transferred to the Eppendorf arena using a 000 paintbrush.

#### 2.4.2 Insect‐only exposure

Insect‐only exposure was carried out to assess the effect of the bioinsecticides when applied directly to the insect only. All *M. persicae*, *B. brassicae*, *D. rapae* adults, mummies and *C. carnea* larvae were individually treated by direct application outside of the containment arena and immediately transferred to their respective untreated assay containment. Mobile stages were transferred using a 000 paintbrush, while parasitoid mummies were moved using storkbill fine‐pointed forceps to avoid additional surface contact.

#### 2.4.3 Residue exposure

Residue exposure was carried out to assess the effect of the bioinsecticides against the pests *M. persicae*, *B. brassicae* and the natural enemies *D. rapae* adults and mummies, and *C. carnea* larvae when exposed to dry residues on the leaf disc. Following application, treated leaf discs were left to dry for a standardised 45 min in a controlled environment room at 20 °C and 60% relative humidity with a 16 h:8 h (light:dark) photoperiod, ensuring that all treatments showed no signs of moisture before being individually transferred to an untreated containment arena using a pair of storkbill fine‐pointed forceps. A single untreated insect was then transferred to the containment arena using a 000 paintbrush.

### 2.5 Statistical analysis

Kaplan–Meier survival curves were generated using the survival package^42^ in R version 4.4.0^40^ to compare overall insect survival between treatment groups and visualise survival patterns. Prior to treatment, any insects that died or leaf discs that deteriorated were replaced to ensure all replicates entered the experiment in a standardised condition; no replicates were excluded after treatment application. For experiments investigating the role of exposure method on survival, log‐rank tests were performed to assess differences in survival distributions. Accelerated failure time (AFT) models were also fitted using the survival package^42^ with a log‐logistic distribution to estimate the direct effect of treatments on insect survival time. Day was included as a factor to account for temporal blocking. Model selection was guided by Akaike's information criterion, and the log‐logistic distribution was selected for the AFT models based on its superior fit, which was confirmed through residual diagnostics conducted using the survminer package.^43^

To assess the effects of treatment and exposure method on emergence of parasitoid mummies, binomial generalised linear models were fitted with emergence (success/failure) as the response variable and treatment as a fixed effect. *Post hoc* pairwise comparisons were performed using estimated marginal means with Holm correction to adjust for multiple testing. Model diagnostics, including checks for overdispersion, uniformity and outliers, were conducted using the DHARMa package.^44^

## 3 RESULTS

### 3.1 Combined exposure

#### 3.1.1 Aphids

Aphid survival differed significantly among treatments for both *M. persicae* (*x* ^2^ = 160, df = 5, *P* < 0.001) and *B. brassicae* (*x* ^2^ = 226, df = 5, *P* < 0.001) (Fig. 2). The positive control, sulfoxaflor, was most effective, causing more than 95% mortality in *M. persicae* and *B. brassicae* within 150 and 120 min, respectively, and was significantly more lethal than all other treatments, as shown by pairwise log‐rank comparisons (*P* < 0.001). Fatty acids and silicone polymers achieved 100% mortality in *B. brassicae* within 300 min but were slower and did not approach full mortality in *M. persicae* (73–83% mortality at 4320 min). Surfactants had a minimal effect on *M. persicae* (10% mortality) and a moderate effect on *B. brassicae* (47% mortality). An AFT model identified that sulfoxaflor most significantly reduced aphid longevity (*β* = −5.07, *P* < 0.001), followed by fatty acids (*β* = −4.62, *P* < 0.001), silicone polymers (*β* = −4.40, *P* < 0.001) and surfactants (*β* = −1.36, *P* = 0.004). Median survival times were lowest for sulfoxaflor (*M. persicae* 150 min, *B. brassicae* 120 min), followed by fatty acids (240, 180 min) and silicone polymers (210, 180 min). The water control did not differ from untreated aphids (*β* = 0.43, *P* = 0.55).

**Figure 2:**
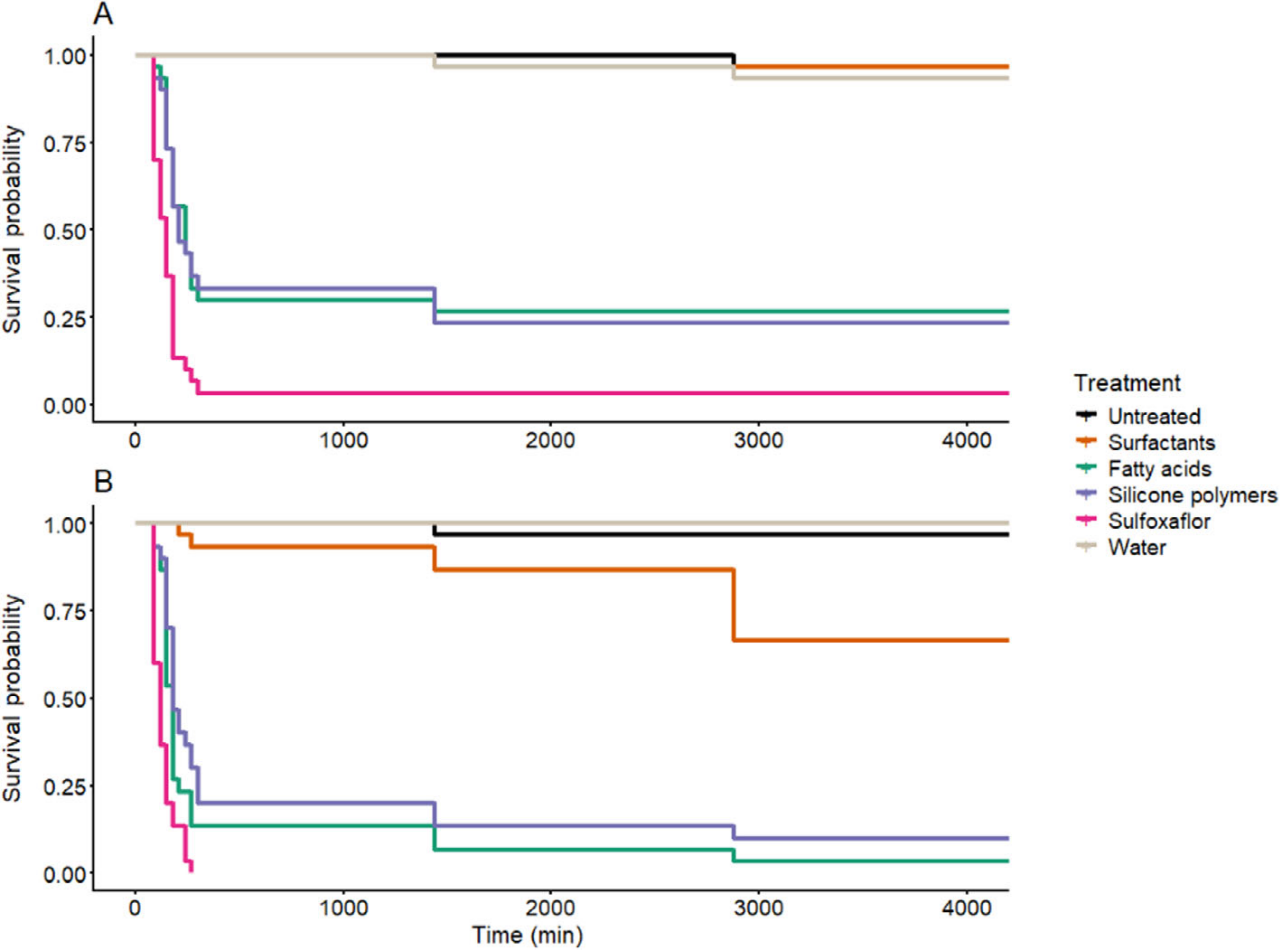
Survival of (A) *Myzus persicae* and (B) *Brevicoryne brassicae* after combined application of negative controls (untreated, water), a synthetic positive control (sulfoxaflor), or one of three bioinsecticides (fatty acids, silicone polymers, surfactants). For both species, survival was significantly affected by treatment (log‐rank test, *P* < 0.001).

#### 3.1.2 Natural enemies

Treatment significantly affected the survival of both *D. rapae* adults (*x* ^2^ = 145, df = 5, *P* < 0.001) and *C. carnea* larvae (*x* ^2^ = 30.4, df = 5, *P* < 0.001) (Fig. 3). For *D. rapae* adults, all insecticide treatments caused significant mortality that reduced survival compared to negative controls (*P* < 0.001). Sulfoxaflor, fatty acids and silicone polymers achieved 100% mortality by 1440 min, while surfactants resulted in 70% mortality in the same timeframe. An AFT model identified that all insecticides significantly reduced survival time, with fatty acids causing the quickest mortality (75 min) followed by silicone polymers (120 min), sulfoxaflor (150 min) then surfactants (180 min). Combined treatment application also affected adult *D. rapae* emergence from aphid mummies (Fig. 4). Fatty acid treatment reduced the predicted probability of emergence to 63%. This represented a significant reduction compared to the untreated control (87%, OR (Odds Ratio) = 3.76, *P* = 0.044) and the water control (90%). Emergence probabilities for silicone polymers (67%), sulfoxaflor (73%) and surfactants (77%) were not statistically different from the controls or fatty acids. Conversely, *C. carnea* larvae were more tolerant to all treatments with silicone polymers (50% mortality at 24 h) and fatty acids (36%) being more lethal than sulfoxaflor (26%), while surfactants had only minor effects (16%). AFT models indicated that silicone polymers (*β* = −5.41, *P* < 0.05), fatty acids (*β* = −4.51, *P* < 0.05) and sulfoxaflor (*β* = −3.81, *P* = 0.03) significantly reduced survival time, whereas surfactants (*P* = 0.11) and the water control (*P* = 0.58) did not. Only silicone polymers reached 50% mortality, with a median survival time of 1440 min.

**Figure 3:**
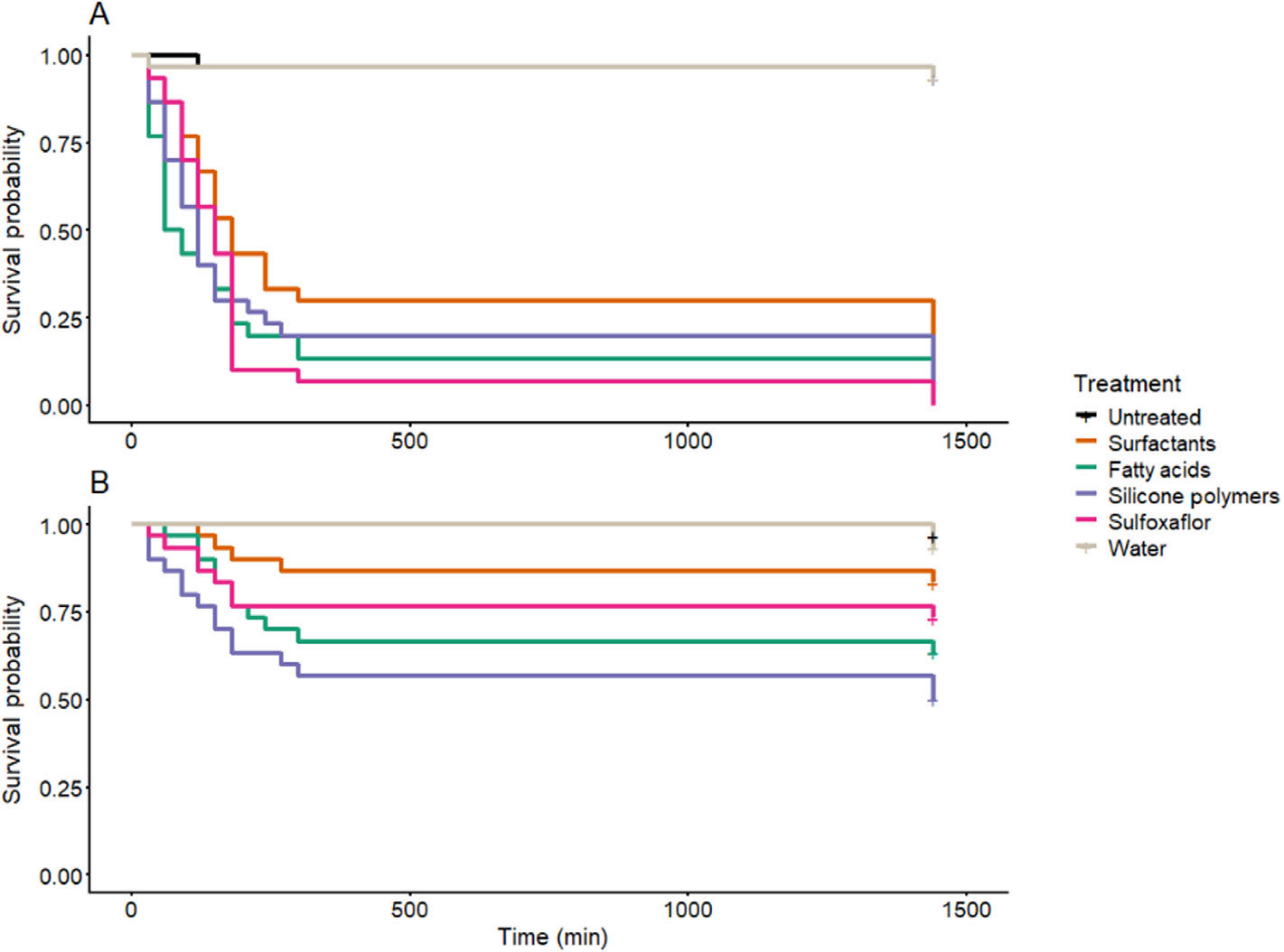
Survival of (A) *Diaeretiella rapae* and (B) *Chrysoperla carnea* after combined application of negative controls (untreated, water), a synthetic positive control (sulfoxaflor), or one of three bioinsecticides (fatty acids, silicone polymers, surfactants). For both species, survival was significantly affected by treatment (log‐rank test, *P* < 0.001).

**Figure 4:**
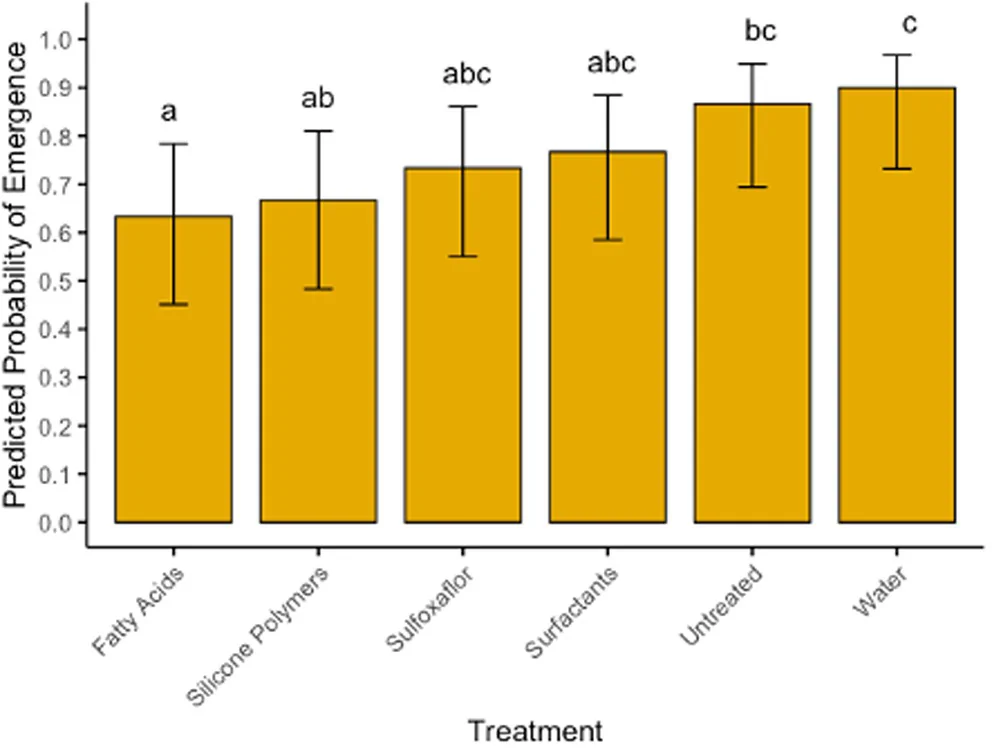
Predicted probability of emergence for *Diaeretiella rapae* from parasitoid mummies after combined application. Bars are model‐estimated means (±95% confidence interval) from a binomial generalised linear model for six treatment groups. Treatments sharing a letter are not significantly different (LSD (Least Significant Difference) pairwise comparisons, *P* < 0.05).

### 3.2 Insect‐only exposure

#### 3.2.1 Aphids

Significant differences in aphid survival were observed among treatments for both *M. persicae* (*x* ^2^ = 103, df = 5, *P* < 0.001) and *B. brassicae* (*x* ^2^ = 178, df = 5, *P* < 0.001) (Fig. 5). Sulfoxaflor was the most effective treatment relative to all others tested (*P* < 0.001), causing more than 85% mortality in *M. persicae* and *B. brassicae* within 300 min, and 90% and 100%, respectively, by the end of the assay (4320 min). Fatty acids and silicone polymers were effective against *B. brassicae*, each achieving 90% mortality at 4320 min, although their impact on *M. persicae* was less pronounced (63% and 53%, respectively). Surfactants provided limited control of *M. persicae* (20% mortality) and moderate control of *B. brassicae* (56% mortality). An AFT model identified sulfoxaflor as the treatment most strongly reducing aphid longevity (*β* = −5.03, *P* < 0.001), followed by silicone polymers (*β* = −3,19, *P* < 0.001), fatty acids (*β* = −3.03, *P* < 0.001) and surfactants (*β* = −1.75, *P* = 0.005). Median survival times were shortest in sulfoxaflor‐treated *M. persicae* (180 min) and *B. brassicae* (150 min), followed by fatty acids (855; 1440 min) and silicone polymers (4320; 1440 min). The water control did not differ from untreated aphids (*β* = 0.99, *P* = 0.35).

**Figure 5:**
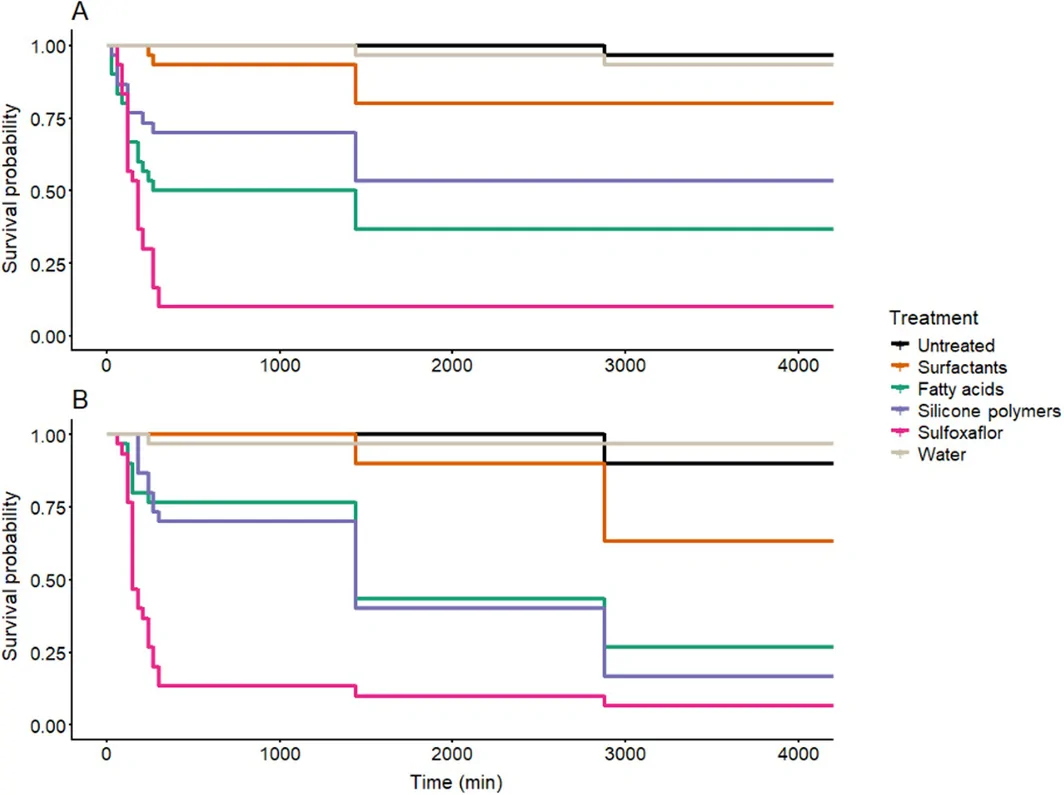
Survival of (A) *Myzus persicae* and (B) *Brevicoryne brassicae* after direct application of negative controls (untreated, water), a synthetic positive control (sulfoxaflor), or one of three bioinsecticides (fatty acids, silicone polymers, surfactants). For both species, survival was significantly affected by treatment (log‐rank test, *P* < 0.001).

#### 3.2.2 Natural enemies

Treatment also significantly affected survival in both *D. rapae* (*x* ^2^ = 178, df = 5, *P* < 0.001) and *C. carnea* (*x* ^2^ = 143, df = 5, *P* < 0.001) (Fig. 6). In *D. rapae*, silicone polymers caused the quickest mortality, with 100% mortality within 30 min, followed by fatty acids (100% at 150 min) and sulfoxaflor (100% at 210 min). All three treatments caused significant mortality compared to negative controls (*P* < 0.001). Mortality associated with surfactants was comparable to that of the negative controls. These findings were confirmed by the AFT model, with median survival time showing silicone polymers and fatty acids as causing the quickest mortality (30 min each) followed by sulfoxaflor (60 min) then surfactants (120 min). Direct application of treatments also affected adult *D. rapae* emergence from aphid mummies (Fig. 7). Treatment with fatty acids, silicone polymers and surfactants all reduced the predicted probability of emergence to 70%. Sulfoxaflor was also observed to cause reduced emergence (76%), although to a lesser extent than the bioinsecticides. Despite this, emergence did not significantly differ between any treatment the untreated control (83%). In contrast, *C. carnea* showed greater tolerance to most treatments with fatty acids (66% mortality at 24 h) and sulfoxaflor (50%) causing moderate mortality, while surfactants had negligible effects (16%). The silicone polymer treatment was significantly more lethal than any other treatment, causing 100% mortality of *C. carnea* in 120 min. This was confirmed by the AFT model, which indicated that silicone polymers (*β* = −5.33, *P* < 0.001), fatty acids (*β* = −4.20, *P* < 0.001) and sulfoxaflor (*β* = −2.971, *P* < 0.001) all significantly reduced survival time, with median survival times of 60, 120 and 210 min, respectively. Surfactants (*P* = 0.36) and the water control (*P* = 0.33) did not differ from the untreated control.

**Figure 6:**
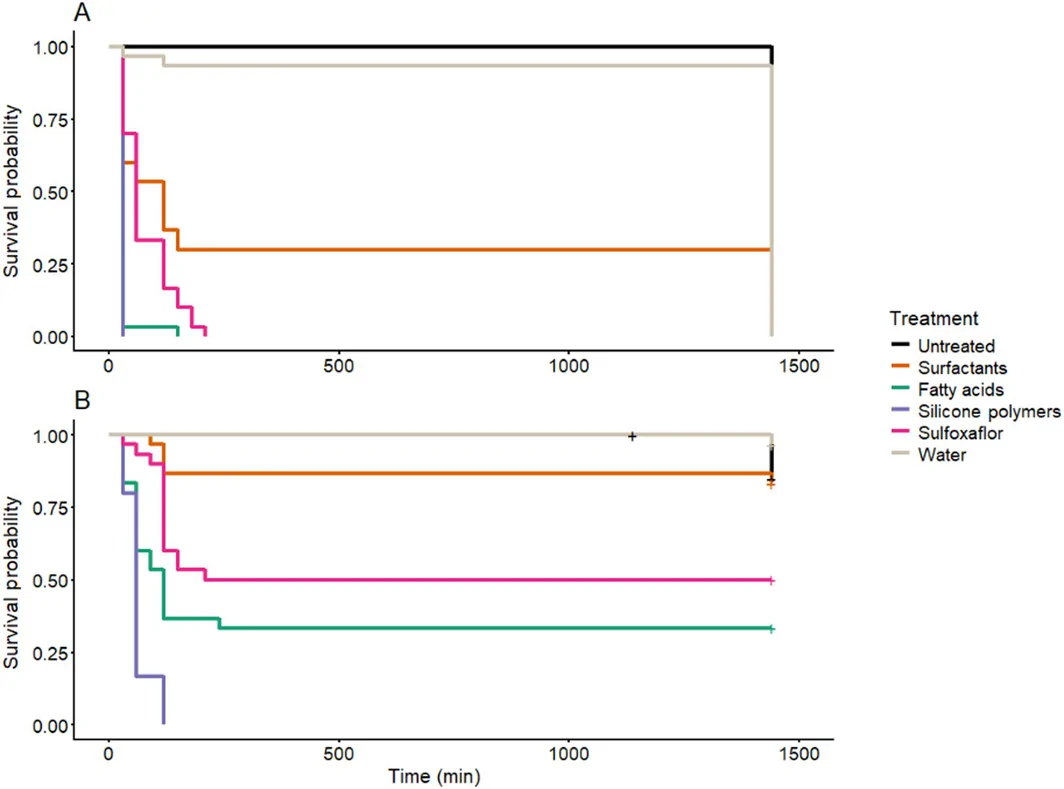
Survival of (A) *Diaeretiella rapae* and (B) *Chrysoperla carnea* after direct application of negative controls (untreated, water), a synthetic positive control (sulfoxaflor), or one of three bioinsecticides (fatty acids, silicone polymers, surfactants). For both species, survival was significantly affected by treatment (log‐rank test, *P* < 0.001).

**Figure 7:**
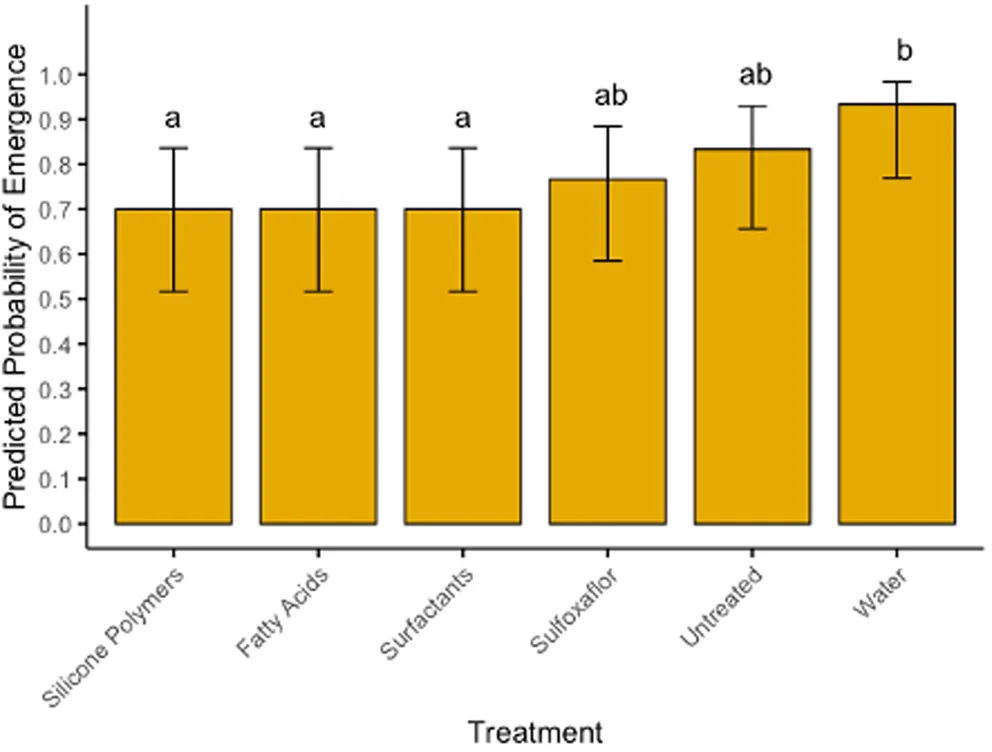
Predicted probability of emergence for *Diaeretiella rapae* from parasitoid mummies after direct application of various treatments. Bars are model‐estimated means (±95% confidence interval) from a binomial generalised linear model for six treatment groups. Treatments sharing a letter are not significantly different (LSD pairwise comparisons, *P* < 0.05).

### 3.3 Residue exposure

#### 3.3.1 Aphids

No mortality was observed for *M. persicae* in any of the bioinsecticide or control treatments, but 100% mortality at 4320 min was recorded following treatment with sulfoxaflor, precluding statistical comparison. In contrast, *B. brassicae* exhibited significant differences in survival across treatments (*x* ^2^ = 256, df = 5, *P* < 0.001) (Fig. 8). Sulfoxaflor residue was significantly more lethal than all other treatments (*P* < 0.001), causing 100% mortality within 4320 min. The fatty acid treatment was observed to cause a more modest reduction in survival (10% mortality at 72 h) followed by silicone polymers (3%) and surfactants (3%), none of which differed from the untreated control (3%). This was confirmed by the AFT model, with sulfoxaflor most significantly reducing aphid longevity (*β* = −4.64, *P* < 0.001) with a median survival time of 240 min.

**Figure 8:**
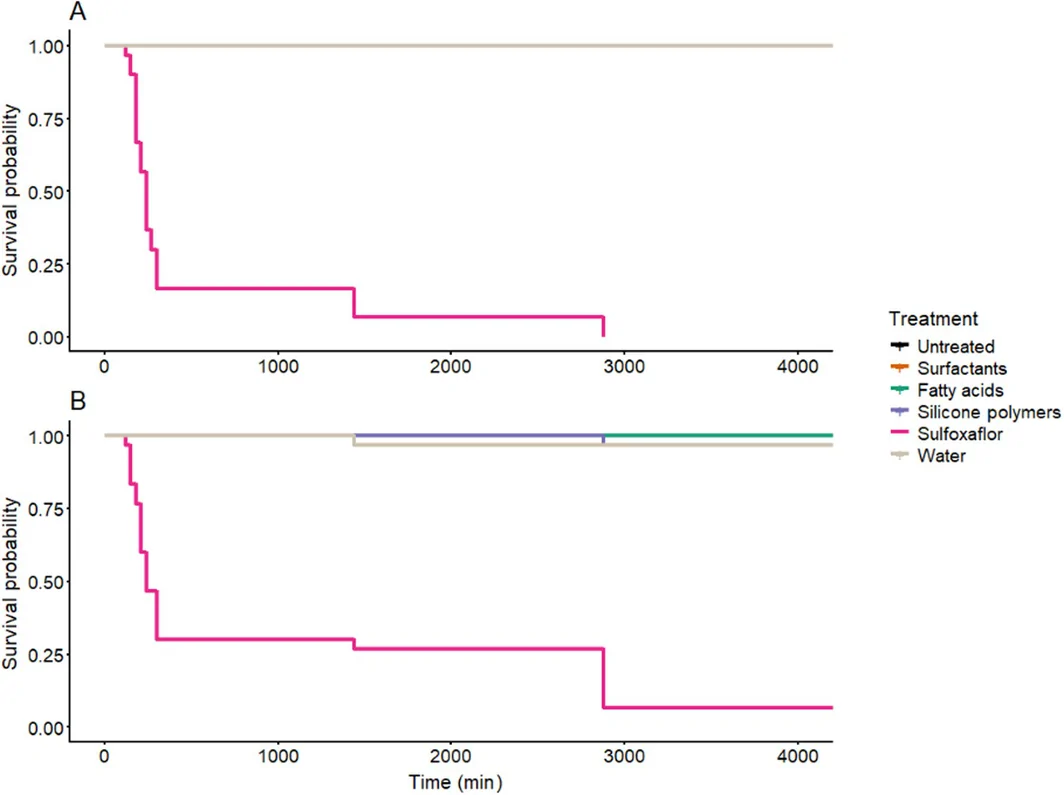
Survival of (A) *Myzus persicae* and (B) *Brevicoryne brassicae* after exposure to residues of negative controls (untreated, water), a synthetic positive control (sulfoxaflor), or one of three bioinsecticides (fatty acids, silicone polymers, surfactants). For both species, survival was significantly affected by treatment (log‐rank test, *P* < 0.001).

#### 3.3.2 Natural enemies

Treatment significantly affected the survival of both *D. rapae* (*x* ^2^ = 91.1, df = 5, *P* < 0.001) and *C. carnea* (*x* ^2^ = 61.6, df = 5, *P* < 0.001) (Fig. 9). For *D. rapae*, sulfoxaflor caused significant mortality (93% in 24 h) that reduced survival compared to all other treatments (*P* < 0.001), while mortality associated with fatty acid residue was more modest (33% in 24 h), although significantly greater than that of the untreated control (*P* = 0.03). No differences were observed between the other treatments and the control groups. An AFT model confirmed both sulfoxaflor (*β* = −5.23, *P* < 0.001) and fatty acid (*β* = −2.68, *P* < 0.05) residues significantly accelerated mortality compared to the untreated control, with a median survival time of 150 min for sulfoxaflor and less than 50% total mortality observed for fatty acids. Exposure to treatment residues did not affect adult *D. rapae* emergence from aphid mummies (Fig. 10). In *C. carnea*, only sulfoxaflor residue was observed to significantly reduce survival (66% mortality in 24 h) compared to all other treatments (≤13%, *P* < 0.001). The AFT model confirmed this, with sulfoxaflor observed to cause the greatest decrease in survival time (*β* = −3.97, *P* < 0.001), with a median survival time of 270 min. No other treatments significantly differed from the untreated control.

**Figure 9:**
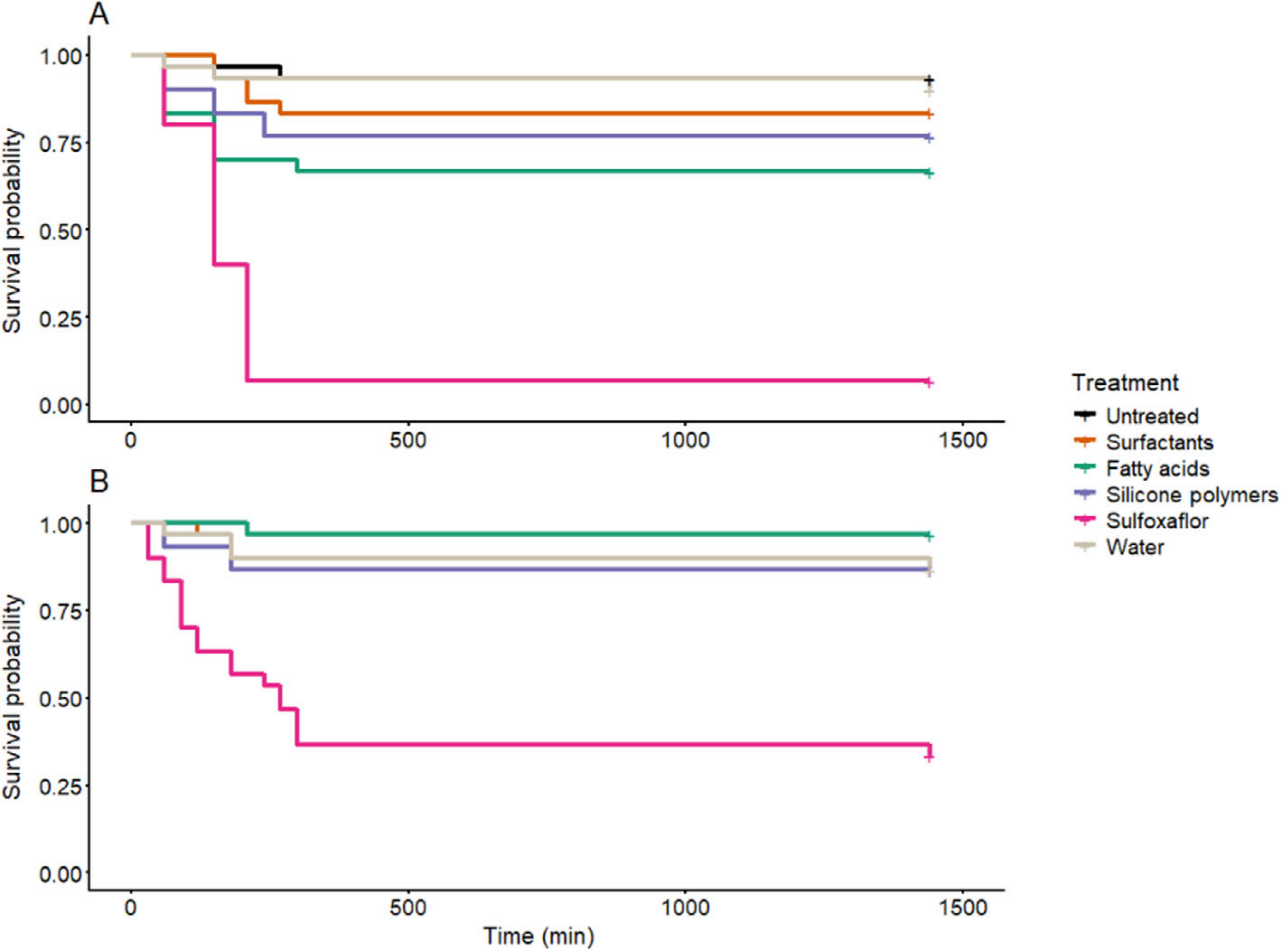
Survival of (A) *Diaeretiella rapae* and (B) *Chrysoperla carnea* after exposure to residues of negative controls (untreated, water), a synthetic positive control (sulfoxaflor), or one of three bioinsecticides (fatty acids, silicone polymers, surfactants). For both species, survival was significantly affected by treatment (log‐rank test, *P* < 0.001).

**Figure 10:**
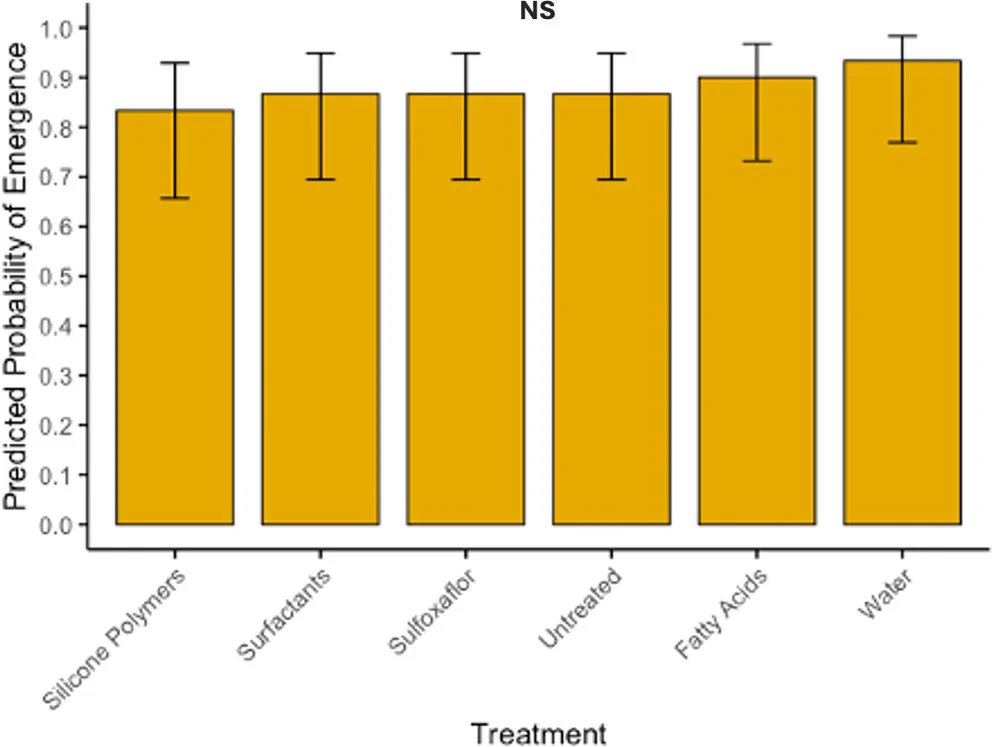
Predicted probability of emergence for *Diaeretiella rapae* from parasitoid mummies after exposure to residues of various treatments. Bars are model‐estimated means (±95% confidence interval) from a binomial generalised linear model for six treatment groups. Treatments sharing a letter are not significantly different (LSD pairwise comparisons, *P* < 0.05). NS, Not significant.

## 4 DISCUSSION AND CONCLUSION

Across both aphid species and natural enemies, efficacy and non‐target risk were strongly dependent on exposure route. Combined exposure, where treatment was applied to both the insect and the localised area, consistently produced higher mortality than insect‐only exposure, likely because simultaneous treatment of both the insect and the surrounding leaf surface maximised contact duration and surface area, thereby enhancing the efficacy of these contact‐dependent products. In contrast, exposure to dried residues resulted in minimal effects across all taxa. This pattern highlights exposure route as a central determinant of bioinsecticide performance and selectivity, and reflects the reliance of these products on direct contact to exert activity.

The bioinsecticides assessed in this study achieved moderate levels of control against both *M. persicae* and *B. brassicae*, with efficacy strongly dependent on direct contact with the insect pest. Among the products tested, fatty acids and silicone polymers were associated with the highest levels of aphid mortality. Whilst peer‐reviewed research investigating the toxicity of these specific formulations to *M. persicae* and *B. brassicae* is limited, insights can be drawn from work on structurally and functionally similar compounds. The fatty acid formulation exhibited a strong dependence on direct or combined exposure, with negligible mortality observed following exposure to dried residues. The minimal residual toxicity observed across all bioinsecticides is consistent with a contact‐dependent mode of action requiring direct interaction with the insect cuticle. This exposure‐route dependence provides strong evidence that the efficacy in the present study was primarily contact‐driven. The fatty acid product contains potassium salts of long‐chain unsaturated carboxylic acids (C7–C20), which have been reported to disrupt or solubilise epicuticular wax layers in soft‐bodied insects, compromising cuticular integrity and accelerating desiccation.^45^, ^46^ Although unsaturated fatty acids have been associated with biochemical effects in insects, having been shown to cause damage to both acetylcholinesterase and cells within the octopaminergic system following peroxidation and subsequent formation of oxidised linoleic acid metabolites,^47^ such mechanisms were not evaluated in this study and cannot be inferred from the observed exposure‐response patterns. The rapid loss of activity once residues had dried, together with the absence of residual toxicity, indicated that any secondary physiological effects are likely contingent on sustained direct contact rather than persistent action. In contrast, silicone polymers are described by the manufacturer as acting through a physical mechanism, forming a flexible polymer ‘net’ on the insect cuticle that restricts mobility and reduces feeding.^27^ This proposed mode of action is consistent with the findings of this study, although the lack of peer‐reviewed evidence verifying either the composition or the mechanism of action limits independent evaluation of its efficacy. Nevertheless, the moderate mortality observed here indicates that silicone polymers may offer useful activity against OSR aphid pests. The surfactant product exhibited the lowest efficacy across exposure routes. Surfactants such as sodium lauryl ether sulphate are widely used to reduce surface tension and enhance coverage, and when applied alone may exert limited insecticidal effects through physical mechanisms such as spiracle blockage or disruption of surface tension.^48^, ^49^, ^50^ However, the comparatively low mortality observed here indicates that surfactants alone are unlikely to provide reliable aphid control in OSR without co‐formulated active ingredients.

In insect‐only exposure assays, mortality associated with fatty acids and silicone polymers was high in *B. brassicae* (90% at 4320 min) but lower in *M. persicae* (63% and 53%, respectively), indicating that species‐specific traits may influence efficacy of contact‐dependent bioinsecticides when exposure is not maximised. Although *B. brassicae* possesses a dense waxy cuticle, it was more susceptible than *M. persicae*, suggesting that cuticle thickness alone does not confer reduced susceptibility, and is consistent with evidence that fatty acids and their potassium salts can disrupt epicuticular wax layers in soft‐bodied insects.^51^, ^52^ In contrast, reduced efficacy against *M. persicae* may reflect behavioural rather than structural differences because this species exhibits greater mobility, which can be amplified in response to detection of synthetic chemical insecticides including pyrethroids, organophosphates, organochlorines and neonicotinoids.^53^, ^54^ Such behaviour, if elicited by physically acting bioinsecticides, could reduce effective contact duration or disrupt retention of fatty acid residues or polymer films, although behavioural responses of *M. persicae* to physically acting bioinsecticides have not been examined. More generally, the requirement for physical contact represents a recognised limitation of these products, particularly for aphids occupying the abaxial leaf surface, and highlights the importance of application coverage and formulation in determining field efficacy.

Although the mortality achieved by the bioinsecticides in this study was not equivalent to the synthetic chemical control, this disparity is not necessarily negative. Reliance on single ‘silver‐bullet’ solutions has historically driven overuse of synthetic chemistries, fostering resistance development and undermining IPM principles.^19^, ^55^ Instead, the value of fatty acids and silicone polymers lies in their potential to diversify the pest management toolkit. The addition of such tools may extend the longevity of existing chemical actives by reducing selection pressure and they are often perceived as having greater compatibility with non‐chemical strategies, such as biological control.^56^ In this respect, moderate mortality is not a limitation but rather a functional component of an integrated approach that balances pest suppression with ecological resilience. A critical dimension of that balance is the compatibility of bioinsecticides with beneficial insects.^57^ The results of this study show that direct or combined contact exposure to fatty acids and silicone polymers caused high mortality in *D. rapae* and moderate mortality in *C. carnea*. Similar findings have been reported elsewhere, with Paspati *et al*.^58^ observing significant mortality of the predatory insect *Nesidiocoris tenuis* Reuter (Hemiptera; Miridae) following topical exposure to fatty acids and organosilicon surfactants have been associated with sublethal effects on bees.^59^, ^60^ These results demonstrate a clear trade‐off: the same exposure routes that produce effective aphid suppression can endanger key natural enemies if direct contact occurs. For IPM systems, such outcomes are undesirable, with conservation biological control being a core pillar of IPM in oilseeds^61^ and effects on pollinators being poorly perceived.^62^

Notably, the residual assays provide evidence of an important ecological safeguard. Across all three bioinsecticide products, dried residues on OSR leaves produced negligible mortality in the natural enemy species tested. Parasitoid emergence from mummies was also unaffected. No significant differences in parasitoid emergence were detected among treatments, including the synthetic chemical sulfoxaflor, indicating that aphid mummies provide effective physical protection to developing parasitoids. This aligns with the intrinsic properties of bioinsecticides, which are thought to lack systemic movement and degrade rapidly after application.^63^, ^64^ Under field conditions, the rate at which bioinsecticide residues degrade is likely to be influenced by environmental and operational factors, including rainfall, ultraviolet exposure, temperature and spray application parameters, all of which can affect contact probability and persistence.^64^ In contrast, synthetic systemic insecticides such as sulfoxaflor remain active in plant tissues for extended periods,^65^, ^66^ exposing natural enemies regardless of their microhabitat within the crop. Some contact‐acting synthetics can exhibit extended residual toxicity, although persistence varies considerably among compounds and environmental conditions.^67^ The comparative brevity of bioinsecticide residues is therefore a considerable advantage for IPM because it reduces the temporal and spatial overlap between pesticide presence and beneficial insect activity. The ecological behaviour of natural enemies further reduces the likelihood of harmful exposure in field conditions. Parasitoids such as *Aphidius colemani* Viereck (Hymenoptera: Braconidae) and *D. rapae* frequently move between crop and non‐crop habitats for nectar, microclimatic buffering and refuge during disturbance.^68^, ^69^, ^70^, ^71^ Such mobility could help them avoid direct exposure during spraying events, returning safely once residues have dried. *Chrysoperla carnea* larvae, although less mobile across habitats, exhibit strong thigmotactic behaviour and often shelter on abaxial surfaces or within structural gaps,^72^ again limiting their likelihood of direct spray contact. While not eliminating risk, these behaviours suggest that mortality observed under intensive laboratory exposures likely overestimates field‐level consequences. Nevertheless, further work is required to quantify behavioural responses of natural enemies to spraying disturbance in real cropping environments.

Taken together, these findings demonstrate that naturally derived products such as bioinsecticides should not be assumed to be inherently benign or universally compatible with IPM simply by virtue of their origin. In this study, fatty acids and silicone polymers were less effective against aphid pests than the synthetic chemical control and caused substantial mortality in beneficial insects under direct exposure, indicating that natural origin does not equate to ecological safety. Their principal advantage lies in their low residual activity, which limits persistence and allows beneficial populations the opportunity to safely recolonise treated areas relatively quickly. As a result, any role for these products in OSR IPM programmes is likely to be conditional on precise, targeted application to pest populations and careful consideration of exposure routes, rather than reliance on residual activity. However, inappropriate or repeated sublethal exposure could still impose selection pressure, reinforcing the need to deploy these products as part of integrated strategies rather than as standalone or repeatedly applied interventions. Further field‐based research is therefore required to define application strategies that balance pest suppression with protection of beneficial arthropods under realistic agronomic conditions.

## CONFLICT OF INTEREST

The authors declare no conflicts of interest.

## Data Availability

The data that support the findings of this study are available from the corresponding author upon reasonable request.
